# Telemedical Interventions for Chronic Obstructive Pulmonary Disease Management: Umbrella Review

**DOI:** 10.2196/33185

**Published:** 2023-02-16

**Authors:** Jin Hean Koh, Lydia Ching Yee Chong, Gerald Choon Huat Koh, Shilpa Tyagi

**Affiliations:** 1 Yong Loo Lin School of Medicine National University of Singapore Singapore Singapore; 2 Ministry of Health Office for Healthcare Transformation Ministry of Health Singapore Singapore

**Keywords:** telemedicine, telehealth, chronic obstructive pulmonary disease

## Abstract

**Background:**

Chronic obstructive pulmonary disease (COPD) is a growing epidemic, with a heavy associated economic burden. Education, physical activity, and pulmonary rehabilitation programs are important aspects of the management of COPD. These interventions are commonly delivered remotely as part of telemedicine interventions. Several systematic reviews and meta-analyses have been conducted to assess the effectiveness of these interventions. However, these reviews often have conflicting conclusions.

**Objective:**

We aim to conduct an umbrella review to critically appraise and summarize the available evidence on telemedicine interventions for the management of COPD.

**Methods:**

In this umbrella review, the MEDLINE, Embase, PsycINFO, and Cochrane databases were searched from inception to May 2022 for systematic reviews and meta-analyses relating to telemedicine interventions for the management of COPD. We compared odds ratios, measures of quality, and heterogeneity across different outcomes.

**Results:**

We identified 7 systematic reviews that met the inclusion criteria. Telemedicine interventions used in these reviews were teletreatment, telemonitoring, and telesupport. Telesupport interventions significantly reduced the number of inpatient days and quality of life. Telemonitoring interventions were associated with significant reductions in respiratory exacerbations and hospitalization rates. Teletreatment showed significant effectiveness in reducing respiratory exacerbations, hospitalization rate, compliance (acceptance and dropout rate), and physical activity. Among studies that used integrated telemedicine interventions, there was a significant improvement in physical activity.

**Conclusions:**

Telemedicine interventions showed noninferiority or superiority over the standard of care for the management of COPD. Telemedicine interventions should be considered as a supplement to usual methods of care for the outpatient management of COPD, with the aim of reducing the burden on health care systems.

## Introduction

Chronic obstructive pulmonary disease (COPD) is a complex chronic respiratory condition, frequently caused by exposure to toxic gases or particles. It is a significant cause of morbidity and death, with heavy economic and social burdens [1,2]. The cost of COPD is €38.6 billion (US $41.3 billion) in the European Union, accounting for some 56% of the cost of respiratory disease [3]. In the United States, the total estimated direct and indirect costs amount to US $52.4 billion [4]. A cornerstone of COPD management is nonpharmacological treatments such as education, physical activity, and pulmonary rehabilitation programs. These interventions may be delivered through physical sessions or remotely.

Digital interventions have the ability to connect patients with health care professionals, enhancing the management of their conditions [5]. For instance, telemedicine care can improve quality of life (QoL) and activity levels and reduce the number of hospitalizations [6,7]. Technology-based interventions can provide convenient accessible means to enhance the exercise capacity of patients, thus educating and motivating healthy lifestyle changes [8]. Internet-mediated interventions may benefit patients with low self-efficacy at baseline by helping them increase physical activity. Similarly, home-based pulmonary rehabilitation programs delivered with remote supervision may facilitate earlier discharge and access to postdischarge care and may be cost-effective [9-11].

Telemedicine is defined as the systematic provision of health care services over physically separate environments via information and communications technology [12]. Telemedicine may be classified as the synchronous or asynchronous transmission of data, remote patient monitoring (telemonitoring), or mobile health, which includes the use of mobile communication devices to deliver targeted messages and education to influence behavioral changes [12]. Telemedicine thus augments traditional methods of managing COPD.

The current COVID-19 pandemic has further brought telemedicine to the forefront of the management of chronic diseases including COPD. Telemedicine has emerged as a safer alternative to usual clinical management in chronic respiratory diseases. The development of novel cheap technologies will increase the number of patients requesting telemedicine services. However, the effectiveness of telemedicine interventions is still unclear [13], with several meta-analyses and systematic reviews reporting conflicting results on several outcome measures due to the poor quality of evidence [14]. Similarly, within the population of patients with COPD, several systematic reviews and meta-analyses have been conducted, although several reviews were unable to draw meaningful conclusions on the overall effect of telemedicine interventions due to the low level of evidence.

Umbrella reviews are reviews of previously published systematic reviews and meta-analyses, adopting a uniform approach to allow for their comparison. Umbrella reviews thus represent one of the highest levels of evidence synthesis currently available [15]. In conducting this review, we took reference from a recent umbrella review by Hailes et al [16].

With rapid uptake and easy access, digital technologies may be considered a potential platform for managing COPD. These technologies may facilitate patient involvement and reduce the burden on health care systems [17]. Given the conflicting evidence and the large number of evidence syntheses evaluating the effectiveness of telemedicine interventions in the management of COPD, the objective of this umbrella review is to synthesize and appraise the large body of evidence in published systematic reviews on the effectiveness of telemedicine interventions in COPD management.

## Methods

### Search Strategy

The authors performed an umbrella review in which information from existing meta-analyses of studies on outcomes of telemedicine for COPD was systematically collected and summarized. A systematic search was conducted on MEDLINE (via PubMed), Embase, PsycINFO, and Cochrane Library for articles relating to telemedicine interventions for COPD from inception up to May 2022. There was no restriction on the date of publication. The search strategy involved a combination of the Medical Subject Headings (MeSH) keywords “pulmonary disease, chronic obstructive” and “telemedicine,” and the non-MeSH term “telehealth.” The full search strategy is available in Multimedia Appendix 1. Only English language articles were included. The references of the included reviews were manually searched. References were managed with Endnote X9 (Clarivate), and duplicates were removed before the title and abstract screening.

### Study Selection and Data Extraction

Two authors (JHK and LCYC) screened the title and abstract of the articles followed by full-text screening, and any disagreements were resolved via discussion with a third author (ST). One author (JHK) extracted data on patient characteristics, the characteristics of interventions, and all outcome data on the effectiveness of COPD management and smoking cessation using a prespecified data extraction form. There were no restrictions on the outcomes to be collected.

### Eligibility Criteria

The inclusion criteria were meta-analyses that reported outcomes following telemedical interventions for the treatment of COPD in patients older than 18 years in all COPD stages, full-text studies, published in a peer-reviewed journal, and written in English. The exclusion criteria were systematic reviews without meta-analysis, reviews of only self-management interventions, narrative reviews, opinion articles, and existing umbrella reviews.

Where more than one meta-analysis reported data for the same outcome, the most recent review that met the inclusion criteria was selected. Older meta-analyses were excluded to avoid the duplication of samples. When two or more meta-analyses reported data for the same outcome and were published within the same year, the meta-analysis with the largest number of primary studies was selected, and the others were excluded.

### Statistical Analysis and Quality Assessment

All effect sizes and CIs were converted into odds ratios (ORs) to enable comparison across outcomes. ORs represented the odds that an outcome would occur given a particular telemedicine intervention compared with the odds of the outcome occurring without telemedicine. The sample sizes are represented as the total sample size (x; x), where x represents the sample size of each primary study included in the systematic review. All included articles were critically appraised independently by two reviewers (JHK and LCYC) using the Assessing the Methodological Quality of Systematic Reviews (AMSTAR) 2 tool [18]. Any disagreements in the quality appraisal were resolved by discussion with the senior author (ST).

## Results

### Summary of Included Articles

**Figure 1:**
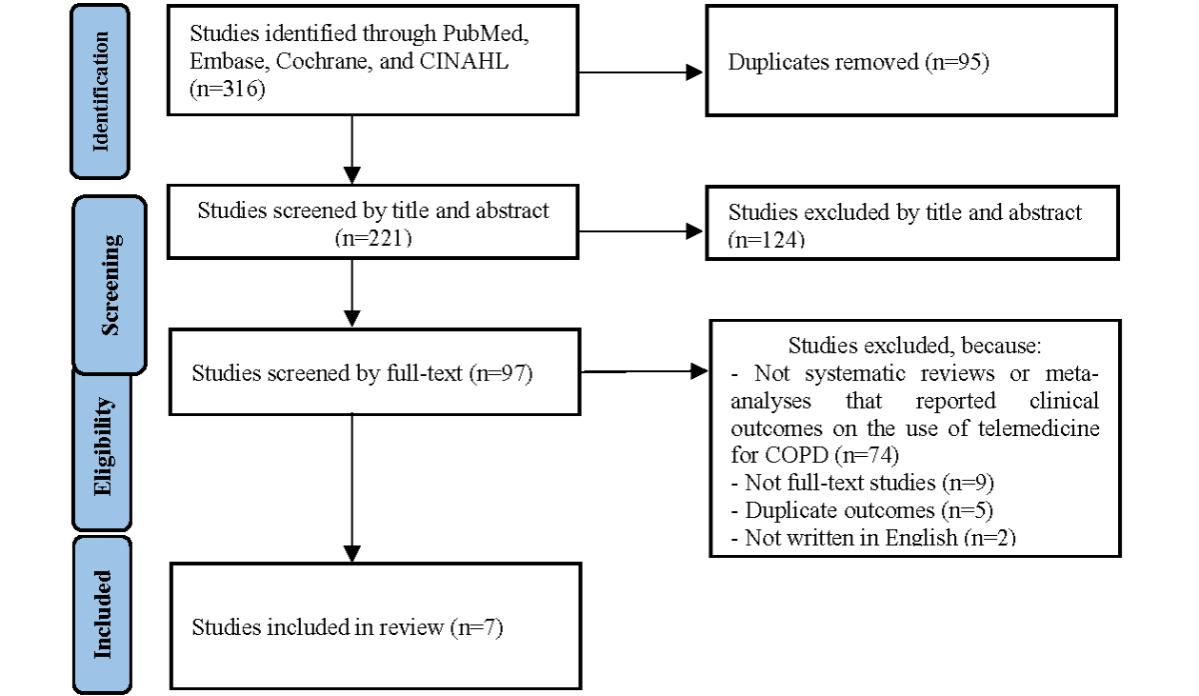
PRISMA (Preferred Reporting Items for Systematic Reviews and Meta-Analyses) flow diagram. COPD: chronic obstructive pulmonary disease.

A total of 221 articles were included in the initial search after the removal of duplicates, of which 97 were selected for full-text review. We identified 7 systematic reviews that met the final inclusion criteria with 7457 participants across 24 outcomes [19-25]. The date range of included systematic reviews was from 2014 to 2022. The number of primary studies in each meta-analysis ranged from 9 to 29, and the number of participants ranged from 587 to 5654. The ORs ranged from 1.44 (95% CI 0.41-4.92) to 0.63 (95% CI 0.51-0.78). Table 1 summarizes the quality assessment of included articles. The aggregate score of the included articles ranged from 6 to 16. The PRISMA flow diagram is shown in Figure 1. Characteristics of the included studies are shown in Table 2, and the summary statistics of the effect on health outcomes are shown in Table 3.

**Table 1:** Assessing the Methodological Quality of Systematic Reviews checklist results and scores.

Study	Outcomes	Q1	Q2	Q3	Q4	Q5	Q6	Q7	Q8	Q9	Q10	Q11	Q12	Q13	Q14	Q15	Q16	Aggregate score
Bonnevie et al [19] 2021	Functional dyspnea, health status, quadriceps force, objective physical activity, subjective physical activity, self-efficacy, withdrawal, adherence	Y	Y	Y	Y	Y	Y	N	Y	Y	N	Y	N	N	N	Y	Y	11
Cruz et al [25] 2014	Mean number of ED^a^ visits	Y	N	Y	Y	N	N	N	Y	N	N	Y	N	N	N	N	Y	6
Hong and Lee [20] 2018	Outpatient visit rates	Y	N	Y	Y	N	Y	N	Y	Y	N	Y	N	N	Y	Y	Y	10
Janjua et al [21] 2021	Exacerbations, adverse events	Y	Y	Y	Y	Y	Y	Y	Y	Y	Y	Y	Y	Y	Y	Y	Y	16
Lu et al [22] 2021	Anxiety, depression, hospitalization (COPD^b^-related), length of hospital stay (all-cause), ED visit rates, mortality	Y	Y	Y	Y	Y	Y	N	Y	Y	N	Y	N	N	Y	N	Y	11
Michaelchuk et al [23] 2022	Exercise capacity, QoL^c^ (short-term), QoL (long-term)	Y	N	Y	Y	Y	Y	N	Y	Y	N	Y	N	N	Y	N	Y	10
Sul et al [24] 2020	Mean number of hospitalizations	Y	N	Y	Y	N	N	N	Y	Y	N	Y	N	N	Y	N	Y	8

^a^ED: emergency department.

^b^COPD: chronic obstructive pulmonary disease.

^c^QoL: quality of life.

**Table 2:** Characteristics of meta-analyses included in this umbrella review.

	Databases searched, n	Outcome	Primary studies, n	Sample size, n^a^	Review (year range)	Country
Bonnevie et al [19] 2021	7	Functional dyspnea	2	152 (103; 49)	2011-2021	Australia
Bonnevie et al [19] 2021	7	Health status	2	354 (37; 317)	2011-2021	Australia
Bonnevie et al [19] 2021	7	Quadriceps force	1	318 (318)	2011-2021	Australia
Bonnevie et al [19] 2021	7	Objective physical activity	2	54 (37; 17)	2011-2021	Australia
Bonnevie et al [19] 2021	7	Subjective physical activity	2	56 (37; 19)	2011-2021	Australia
Bonnevie et al [19] 2021	7	Self-efficacy	2	123 (37; 86)	2011-2021	Australia
Bonnevie et al [19] 2021	7	Withdrawal rates	3	327 (90; 103; 134)	2011-2021	Australia
Bonnevie et al [19] 2021	7	Adherence	2	224 (90; 134)	2011-2021	Australia
Cruz et al [25] 2014	4	Mean number of ED^b^ visits	2	160 (45; 115)	2006-2012	Portugal
Hong and Lee [20] 2018	3	Outpatient visit rates	3	436 (343; 48; 45)	2006-2017	Korea
Janjua et al [21] 2021	4	Exacerbations	3	955 (319; 166; 470)	2009-2020	UK
Janjua et al [21] 2021	4	Adverse events	2	485 (319; 166)	2009-2020	UK
Janjua et al [21] 2021	4	QoL^c^ (long-term)	2	203 (62; 141)	2009-2020	UK
Lu et al [22] 2021	3	Anxiety	2	444 (400; 44)	2006-2019	China
Lu et al [22] 2021	3	Depression	2	444 (400; 44)	2006-2019	China
Lu et al [22] 2021	3	Hospitalization (all-cause)	4	772 (155; 281; 307; 29)	2006-2019	China
Lu et al [22] 2021	3	Hospitalization (COPD^d^-related)	7	1281 (155; 29; 110; 281; 67; 106; 334)	2006-2019	China
Lu et al [22] 2021	3	Length of hospital stay (all-cause)	6	1073 (256; 29; 281; 334; 42; 131)	2006-2019	China
Lu et al [22] 2021	3	Length of hospital stay (COPD-related)	7	2201 (229; 281; 62; 256; 334; 312; 727)	2006-2019	China
Lu et al [22] 2021	3	ED visit rates	6	1099 (44; 62; 29; 281; 106; 577)	2006-2019	China
Lu et al [22] 2021	3	Mortality	11	2307 (155; 44; 62; 256; 281; 67; 334; 42; 319; 470; 279)	2006-2019	China
Michaelchuk et al [23] 2022	5	Exercise capacity	7	435 (92; 65; 81; 44; 20; 36; 97)	2008-2021	Portugal
Michaelchuk et al [23] 2022	5	QoL (short-term)	4	290 (92; 65; 20; 97)	2008-2021	Portugal
Sul et al [24] 2020	4	Mean number of hospitalizations	5	517 (254; 80; 45; 36; 102)	2008-2018	Korea

^a^(x; x): sample size for each primary study.

^b^ED: emergency department.

^c^QoL: quality of life.

^d^COPD: chronic obstructive pulmonary disease.

**Table 3:** Effect of telemedicine on health outcomes.

Outcome	Odds ratio^a^ (95% CI)	*I*² (%)	Direction of change
Mean number of emergency department visits [25]	1.44 (0.41-4.92)	75	No change
Functional dyspnea [19]	1.38 (1.00-1.90)	0	Decreased with control
Self-efficacy [19]	1.38 (1.00-1.89)	NR^b^	Increased with control
Objective physical activity [19]	1.37 (0.85-2.22)	NR	No change
Subjective physical activity [19]	1.36 (0.84-2.18)	NR	No change
Exercise capacity [23]	1.18 (0.99-1.40)	46	No change
Withdrawal rates [19]	1.1 (0.30-3.40)	NR	No change
Anxiety [22]	1.02 (0.86-1.21)	0	No change
Depression [22]	1.01 (0.85-1.19)	0	No change
Quadriceps force [19]	1 (0.88-1.31)	NR	No change
Adherence [19]	1 (0.90-1.30)	NR	No change
Exacerbations [21]	0.98 (0.74-1.28)	0	No change
Length of hospital stay (COPD^c^-related) [22]	0.97 (0.90-1.05)	41	No change
Mean number of hospitalizations [24]	0.96 (0.82-1.12)	3	No change
Quality of life (long-term) [21]	0.95 (0.74-1.22)	0	No change
Hospitalization (all-cause) [22]	0.92 (0.78-1.08)	27	No change
Adverse events [21]	0.91 (0.62-1.33)	0	No change
Emergency department visit rates [22]	0.89 (0.80-1.00)	95	No change
Length of hospital stay (all-cause) [22]	0.87 (0.78-0.97)	16	No change
Outpatient visit rates [20]	0.84 (0.69-1.03)	0	No change
Health status [19]	0.83 (0.69-1.00)	NR	No change
Hospitalization (COPD-related) [22]	0.74 (0.60-0.92)	73	Decreased with telemedical interventions
Mortality [22]	0.71 (0.54-0.93)	40	Decreased with telemedical interventions
Quality of life (short-term) [23]	0.63 (0.51-0.78)	38	Increased with telemedical interventions

^a^Odds ratios: values <1 favor telemedical interventions and values >1 favor control.

^b^NR: not reported.

^c^COPD: chronic obstructive pulmonary disease.

### Mean Number of Emergency Department Visits

The mean number of emergency department (ED) visits was assessed in a systematic review of two primary studies including 160 (n=45; n=115) patients [25]. Telemedicine interventions were not associated with a significant increase in the mean number of ED visits. The OR of ED visit rates was 1.44 (95% CI 0.41-4.92).

### Functional Dyspnea

Functional dyspnea was assessed in a systematic review of two primary studies including 152 (n=103; n=49) patients [19]. Telemedicine interventions were not associated with a significant increase in functional dyspnea. The OR of functional dyspnea was 1.38 (95% CI 1.00-1.90).

### Self-efficacy

Self-efficacy was assessed in a systematic review of two primary studies including 123 (n=37; n=86) patients [19]. Telemedicine interventions were not associated with a significant increase in self-efficacy. The OR of self-efficacy was 1.38 (95% CI 1.00-1.89).

### Objective Physical Activity

Objective physical activity was assessed in a systematic review of two primary studies including 54 (n=37; n=17) patients [19]. Telemedicine interventions were not associated with a significant increase in objective physical activity. The OR for objective physical activity was 1.37 (95% CI 0.85-2.22).

### Subjective Physical Activity

Subjective physical activity was assessed in a systematic review of two primary studies including 56 (n=37; n=19) patients [19]. Telemedicine interventions were not associated with a significant increase in subjective physical activity. The OR for objective physical activity was 1.36 (95% CI 0.84-2.18).

### Exercise Capacity

Exercise capacity was assessed in a systematic review of 7 primary studies including 435 (n=92; n=65; n=81; n=44; n=20; n=36; n=97) patients [23]. Telemedicine interventions were not associated with a significant increase in exercise capacity. The OR for exercise capacity was 1.18 (95% CI 0.99-1.40).

### Withdrawal Rates

Withdrawal rates were assessed in a systematic review of 3 primary studies including 327 (n=90; n=103; n=134) patients [19]. Telemedicine interventions were not associated with a significant increase in withdrawal rates. The OR for exercise capacity was 1.1 (95% CI 0.30-3.40).

### Anxiety

Anxiety was assessed in a systematic review of two primary studies including 444 patients [22]. Telemedicine interventions were not associated with a significant increase in anxiety. The OR for anxiety was 1.02 (95% CI 0.86-1.21).

### Depression

Depression was assessed in a systematic review of 2 primary studies including 444 (n=400; n=44) patients [22]. Telemedicine interventions were not associated with a significant increase in depression. The OR for depression was 1.01 (95% CI 0.85-1.19).

### Quadriceps Force

Quadriceps force was assessed in a systematic review of 1 primary study including 318 patients [19]. Telemedicine interventions were not associated with a significant increase in quadriceps force. The OR for quadriceps force was 1.00 (95% CI 0.88-1.31).

### Adherence

Adherence was assessed in a systematic review of 2 primary studies including 224 (n=90; n=134) patients [19]. Telemedicine interventions were not associated with a significant increase in adherence. The OR for adherence was 1.00 (95% CI 0.90-1.30).

### Exacerbation Rates

Exacerbation rates were assessed in a systematic review of 3 primary studies including 955 (n=319; n=166; n=470) patients [21]. Telemedicine interventions were not associated with a significant increase in exacerbation rates. The OR for exacerbation rates was 0.98 (95% CI 0.74-1.28).

### Length of Stay (COPD-Related)

Length of stay (COPD-related) was assessed in a systematic review of 7 primary studies including 2201 (n=229; n=281; n=62; n=256; n=334; n=312; n=727) patients [22]. Telemedicine interventions were not associated with a significant increase in length of stay. The OR for the length of stay was 0.97 (95% CI 0.90-1.05).

### Mean Number of Hospitalizations

The mean number of hospitalizations was assessed in a systematic review of 5 primary studies including 517 (n=254; n=80; n=45; n=36; n=102) patients [24]. Telemedicine interventions were not associated with a significant increase in the mean number of hospitalizations. The OR for the mean number of hospitalizations was 0.96 (95% CI 0.82-1.12).

### Quality of Life (Long-term)

Long-term QoL was assessed in a systematic review of 2 primary studies including 203 (n=62; n=141) patients [23]. Telemedicine interventions were not associated with a significant increase in long-term QoL. The OR for long-term QoL was 0.95 (95% CI 0.74-1.22).

### Hospitalization (All-Cause)

All-cause hospitalization was assessed in a systematic review of 4 primary studies including 772 (n=155; n=281; n=307; n=29) patients [22]. Telemedicine interventions were not associated with a significant increase in all-cause hospitalization. The OR for all-cause hospitalization was 0.92 (95% CI 0.78-1.08).

### Adverse Event Rates

Adverse event rates were assessed in a systematic review of 2 primary studies including 485 (n=319; n=166) patients [21]. Telemedicine interventions were not associated with a significant increase in adverse event rates. The OR for adverse event rates was 0.91 (95% CI 0.62-1.33).

### ED Visit Rates

ED visit rates were assessed in a systematic review of 6 primary studies including 1099 (n=44; n=62; n=29; n=281; n=106; n=577) patients [22]. Telemedicine interventions were not associated with a significant increase in ED visit rates. The OR for ED visit rates was 0.89 (95% CI 0.80-1.00).

### Length of Hospital Stay (All-Cause)

All-cause length of hospital stay was assessed in a systematic review of 6 primary studies including 1073 (n=256; n=29; n=281; n=334; n=42; n=131) patients [22]. Telemedicine interventions were associated with a significant reduction in all-cause length of hospital stay. The OR for all-cause length of hospital stay was 1.00 (95% CI 0.78-0.97).

### Outpatient Visit Rates

Outpatient visit rates were assessed in a systematic review of 3 primary studies including 436 (n=343; n=48; n=45) patients [20]. Telemedicine interventions were not associated with a significant increase in outpatient visit rates. The OR for outpatient visit rates was 1.00 (95% CI 0.69-1.03).

### Health Status

Health status was assessed in a systematic review of 2 primary studies including 354 (n=37; n=317) patients [19]. Telemedicine interventions were not associated with a significant change in health status. The OR for health status was 0.83 (95% CI 0.69-1.00).

### Hospitalization (COPD-Related)

COPD-related hospitalization rates were assessed in a systematic review of 7 primary studies including 1281 (n=155; n=29; n=110; n=281; n=67; n=106; n=334) patients [22]. Telemedicine interventions were associated with a significant reduction in COPD-related hospitalization rates. The OR for COPD-related hospitalization rates was 0.74 (95% CI 0.60-0.92).

### Mortality

Mortality rates were assessed in a systematic review of 11 primary studies including 2307 (n=155; n=44; n=62; n=256; n=281; n=67; n=334; n=42; n=319; n=470; n=279) patients [22]. Telemedicine interventions were associated with a significant reduction in mortality rates. The OR for mortality rates was 0.71 (95% CI 0.54-0.93).

### Quality of Life (Short-term)

Short-term QoL was assessed in a systematic review of 4 primary studies including 290 (n=92; n=65; n=20; n=97) patients [23]. Telemedicine interventions were not associated with a significant increase in short-term QoL. The OR for short-term QoL was 0.63 (95% CI 0.51-0.78).

## Discussion

### Principal Findings

In this umbrella review of 24 outcomes of telemedicine interventions for COPD, we summarized the evidence from 8 systematic reviews, including 7457 participants from across 165 primary studies [19-25]. This is, to the best of our knowledge, the first umbrella review to summarize the effects of telemedicine interventions for the management of COPD. The impact of telemedicine-supported interventions for COPD was consistent, with all included studies reporting either significant improvements or noninferiority of telemedicine interventions as compared to usual care. No reviews reported a negative impact of interventions using telemedicine for COPD management.

Across most of the outcomes, telemedicine interventions did not appear to provide a significant benefit over usual care. However, telemedicine interventions were associated with significant reductions in mortality rates, COPD-related hospitalization rates, and short-term QoL. A possible explanation for these results is that telemedicine interventions were designed specifically for detecting changes in a patient’s COPD status and may be less sensitive to changes in other domains. As such, the benefit of telemedicine interventions was apparent only in COPD-related hospitalization rates and not all-cause hospitalization rates.

### Comparison With Prior Work

The differing results for QoL could arise from the different modalities used to measure QoL. Tools such as the St George’s Respiratory Questionnaire and COPD Assessment Test are specific to COPD, while tools such as the 36-Item Short Form Survey are more generic. Given the severely impaired lung function of patients with COPD at baseline, it is expected that there is a smaller relative change compared with baseline, which may only be captured by more sensitive COPD-specific questionnaires. Taken together, it appears that, while not consistently superior to usual care, telemedicine is a safe alternative mode for the delivery of chronic disease management of COPD.

Several of the included reviews have approached telemedicine interventions in a hybrid manner, combining various kinds of telemedicine interventions including telemonitoring, telerehabilitation, and teleconsultations. However, there were few outcomes reported among reviews that examined the effect of two or more telemedicine interventions. Hence, we were unable to draw meaningful conclusions between one form versus multiple forms of telemedicine interventions. Further empirical studies or randomized controlled trials (RCTs) are needed to confirm the superiority of hybrid telemedicine interventions over one form of intervention only and to compare in a head-to-head manner the effectiveness of each type of intervention.

Patients with COPD are a highly heterogeneous population with different phenotypes. The severity of COPD (mild to very severe) and the age of participants (65-72 years) varied among the studies included, which made it difficult to ascertain which COPD patient subgroups would benefit from single digital interventions or multicomponent interventions in terms of our prespecified primary outcomes. This may make it challenging for health care services to implement reviewed strategies in a tailored manner in practice.

COPD has a high economic burden, with exacerbations accounting for the greatest proportion of the total COPD burden on the health care system. The severity of COPD is proportional to the cost of care, particularly for hospitalization and ambulatory oxygen costs. Furthermore, pulmonary rehabilitation and education are important elements of the management of COPD. Home-based programs have demonstrated equivalence to hospital-based programs in RCTs, provided the frequency and intensity are equivalent [26-28]. Therefore, embedding telemedicine interventions or rehabilitation programs into traditional management plans could help alleviate the burden on health care systems.

This review has highlighted several possible areas for further research. Clear descriptions of intervention components are needed to allow for classification and grouping to reduce heterogeneity in future systematic reviews and meta-analyses. RCTs of telemedicine interventions may also be synthesized through a network meta-analysis, provided there are sufficient data from studies in the future. Future clinical trials should include a cost analysis in their reporting to provide financial insights related to the implementation of the intervention [29]. The lack of detailed data on program costs and health care service savings hampers decision-making on the uptake of telemedicine interventions. The safety of telemedical interventions could not be accurately evaluated in this review due to the lack of systematic reviews assessing this outcome. Therefore, further studies may be warranted to address this pertinent outcome measure.

### Limitations

A key strength of this umbrella review is it being a review of previously published systematic reviews or meta-analyses; they represent one of the highest levels of evidence synthesis currently available [15]. It summarizes the large body of evidence available on telehealth interventions for COPD management. By searching five databases, we were able to identify a large number of reviews for this evidence synthesis. We have also limited the included studies to only systematic reviews and meta-analyses of RCTs to ensure the highest quality of evidence [30]. However, there are limitations inherent to the umbrella review methodology. By relying on the findings of systematic reviews, the evidence is one step removed from empirical evidence and thus reliant on the interpretation of review authors. The impact of the intensity of the interventions is also limited to a few systematic reviews. Such an umbrella review is thus well suited to forming an overview of the topic but loses the granularity and detail of the evidence. The quality of evidence of individual primary studies in the included reviews could not be analyzed as the quality of the included reviews was assessed as a whole using the AMSTAR 2 tool. Another limitation of this review is not having two reviewers independently performing data extraction, and there was no protocol published prior to this review. The inclusion of only reviews published in English may also have contributed to publication bias. However, developing a comprehensive search strategy and screening multiple databases would have alleviated this limitation.

### Conclusion

Although telemedicine interventions were not consistently found to be superior to usual care, no reviews reported any negative effects, suggesting that telemedicine is a safe alternative mode of delivery for the management of a chronic disease.

In the current COVID-19 pandemic, the use of digital interventions has increased significantly, with a greater emphasis on infection control and a concomitant rapid evolution of technology as a result. While telemedicine itself may not be sufficient to achieve significantly better outcomes for patients, these interventions could supplement traditional modes of COPD care to provide remote health care that is personalized and tailored to the individual’s needs.

## Appendix

Multimedia Appendix 1 Search strategy.

## Abbreviations

AMSTAR: Assessing the Methodological Quality of Systematic Reviews
COPD: chronic obstructive pulmonary disease
ED: emergency department
MeSH: Medical Subject Headings
OR: odds ratio
QoL: quality of life
RCT: randomized controlled trial
