# Supplementary material for: Telemedical Interventions for Chronic Obstructive Pulmonary Disease Management: Umbrella Review
Source: J Med Internet Res. 2023 Feb 16;25:e33185. doi: 10.2196/33185 (PMC9982717; doi:10.2196/33185)
Supplement: Multimedia Appendix 1 [file jmir_v25i1e33185_app1.docx]

**Multimedia Appendix 1. Search strategy.**

Search strategy for MEDLINE

1. exp pulmonary disease, chronic obstructive/ or (chronic airflow obstruction or airflow obstruction, chronic or COAD or COPD or 'chronic obstructive pulmonary disease*' or chronic obstructive airway disease or chronic obstructive lung disease).tw.exp Pulmonary Disease, Chronic Obstructive/
2. exp telemedicine/ or (telemedicine or telehealth or eHealth or mHealth or mobile health).tw.
3. 1 and 2
4. ((systematic and review) or 'systematic meta-review' or (systematic search and review) or (meta and (analysis or synthesis or regression)) or 'meta-analys*' or 'meta-regression' or 'meta-synthesis').tw.
5. 3 and 4

Search strategy for Embase

1. 'chronic obstructive lung disease'/exp OR 'chronic obstructive lung disease' OR (chronic:ti,ab,kw AND (airflow:ti,ab,kw OR airway:ti,ab,kw) AND obstruction:ti,ab,kw) OR (chronic:ti,ab,kw AND obstructive:ti,ab,kw AND (bronchopulmonary:ti,ab,kw OR pulmonary:ti,ab,kw OR respiratory:ti,ab,kw OR lung:ti,ab,kw) AND (disease:ti,ab,kw OR disorder:ti,ab,kw)) OR copd:ti,ab,kw
2. 'telehealth'/exp OR 'telemedicine'/exp OR ((tele:ti,ab,kw OR virtual:ti,ab,kw) AND medicine:ti,ab,kw) OR 'e-health':ti,ab,kw OR ehealth:ti,ab,kw OR 'tele-health':ti,ab,kw
3. #1 AND #2
4. systematic:ti,ab,kw AND review:ti,ab,kw OR 'systematic meta-review':ti,ab,kw OR ('systematic search':ti,ab,kw AND review:ti,ab,kw) OR (meta:ti,ab,kw AND (analysis:ti,ab,kw OR synthesis:ti,ab,kw OR regression:ti,ab,kw)) OR 'meta-analys*':ti,ab,kw OR 'meta-regression':ti,ab,kw OR 'meta-synthesis':ti,ab,kw
5. #3 AND #4

Search strategy for Cochrane

1. [mh "pulmonary disease, chronic obstructive"] OR ("chronic airflow obstruction":ti,ab OR "airflow obstruction, chronic":ti,ab OR COAD:ti,ab OR COPD:ti,ab OR ("'chronic obstructive pulmonary" NEXT disease*'):ti,ab OR "chronic obstructive airway disease":ti,ab OR "chronic obstructive lung disease":ti,ab)
2. [mh telemedicine] OR (telemedicine:ti,ab OR telehealth:ti,ab OR eHealth:ti,ab OR mHealth:ti,ab OR "mobile health":ti,ab)
3. #1 AND #2

Search strategy for PsycINFO

1. ("chronic airflow obstruction" or "airflow obstruction, chronic" or COAD or COPD or "'chronic obstructive pulmonary disease*'" or "chronic obstructive airway disease" or "chronic obstructive lung disease").ti,ab.
2. exp telemedicine/ or (telemedicine or telehealth or eHealth or mHealth or "mobile health").ti,ab.
3. ((systematic and review) or "'systematic meta-review'" or ("systematic search" and review) or (meta and (analysis or synthesis or regression)) or 'meta-analys*' or 'meta-regression' or 'meta-synthesis').ti,ab.
4. 1 and 2 and 3
